# Impact of the MICA-129Met/Val Dimorphism on NKG2D-Mediated Biological Functions and Disease Risks

**DOI:** 10.3389/fimmu.2016.00588

**Published:** 2016-12-12

**Authors:** Antje Isernhagen, Dörthe Malzahn, Heike Bickeböller, Ralf Dressel

**Affiliations:** ^1^Institute of Cellular and Molecular Immunology, University Medical Center Göttingen, Göttingen, Germany; ^2^Institute of Genetic Epidemiology, University Medical Center Göttingen, Göttingen, Germany; ^3^DZHK (German Center for Cardiovascular Research), Partner Site Göttingen, Göttingen, Germany

**Keywords:** NK cells, T cells, activating NK receptor, costimulation, single-nucleotide polymorphism, autoimmune diseases, cancer, hematopoietic stem cell transplantation

## Abstract

The major histocompatibility complex (MHC) class I chain-related A (*MICA*) is the most polymorphic non-classical MHC class I gene in humans. It encodes a ligand for NKG2D (NK group 2, member D), an activating natural killer (NK) receptor that is expressed mainly on NK cells and CD8^+^ T cells. The single-nucleotide polymorphism (SNP) rs1051792 causing a valine (Val) to methionine (Met) exchange at position 129 of the MICA protein is of specific interest. It separates MICA into isoforms that bind NKG2D with high (Met) and low affinities (Val). Therefore, this SNP has been investigated for associations with infections, autoimmune diseases, and cancer. Here, we systematically review these studies and analyze them in view of new data on the functional consequences of this polymorphism. It has been shown recently that the MICA-129Met variant elicits a stronger NKG2D signaling, resulting in more degranulation and IFN-γ production in NK cells and in a faster costimulation of CD8^+^ T cells than the MICA-129Val variant. However, the MICA-129Met isoform also downregulates NKG2D more efficiently than the MICA-129Val isoform. This downregulation impairs NKG2D-mediated functions at high expression intensities of the MICA-Met variant. These features of the MICA-129Met/Val dimorphism need to be considered when interpreting disease association studies. Particularly, in the field of hematopoietic stem cell transplantation, they help to explain the associations of the SNP with outcome including graft-versus-host disease and relapse of malignancy. Implications for future disease association studies of the MICA-129Met/Val dimorphism are discussed.

## Introduction

The major histocompatibility complex (MHC) class I chain-related A (*MICA*) is the most polymorphic non-classical MHC class I gene in humans, and 105 alleles are known encoding for 82 protein variants (http://www.ebi.ac.uk/imgt/hla/, release 3.25.0). *MICA* is encoded within the human leukocyte antigen (HLA) complex close to HLA-B (1, 2). The protein structure is similar to classical class I molecules, but MICA is not associated with β2-microglobulin and does not present peptides. MICA is constitutively expressed only on a few cell types, including gastrointestinal epithelium, but is induced due to cellular and genotoxic stress (3, 4), malignant transformation, or virus infection (5, 6). MICA is a ligand for NKG2D (NK group 2, member D), an activating natural killer (NK) receptor encoded by the *KLRK1* gene (7). NKG2D is expressed on most human NK cells, CD8^+^ αβ T cells, γδ T cells, iNKT cells, and subsets of effector or memory CD4^+^ T cells (8, 9). On NK cells, NKG2D signaling elicits killing of target cells (10) and secretion of IFN-γ (11). On CD8^+^ αβ T cells, NKG2D provides a costimulatory signal to activate naïve cytotoxic T lymphocytes (12). NKG2D contributes to the elimination of tumor cells (13) and plays a role in the defense against pathogens (14, 15). In addition to MICA, MICB and the UL16-binding proteins (ULBP) encoded by the retinoic acid early transcript 1 (*RAET1*) family function as ligands for NKG2D. *MICB* is also very polymorphic with 42 alleles encoding 28 protein variants (http://www.ebi.ac.uk/imgt/hla/, release 3.25.0). The *RAET1* gene family is localized on chromosome 6 outside the HLA complex and six loci encode functional proteins (16). *RAET1* genes are less polymorphic than *MICA* and *MICB*.

Polymorphisms of *MICA* have been investigated for their role in infections, autoimmune diseases, and cancer (17–21). The single-nucleotide polymorphism (SNP) rs1051792 (G/A) causing a valine (Val) to methionine (Met) exchange at position 129 in the α2 domain of the MICA protein has gained specific interest. It separates *MICA* alleles into two groups (22). MICA isoforms containing a methionine at position 129 bind NKG2D with high affinity, whereas those with a valine bind NKG2D with low affinity. High-affinity alleles include MICA*001, *002, *007, and *017; among the low-affinity alleles are MICA*004, *006, *008, *009, and *010 (23). Due to its functional consequences, the MICA-129Met/Val dimorphism has been investigated in several disease association studies. Here, we review these studies in view of new data on the functional consequences of this amino acid variation elicited after binding to NKG2D.

## MICA-129Met/Val Disease Association Studies

In September 2016, we searched Pubmed for MICA-129Met/Val disease association studies using the key words rs1051792, MICA-129, MICA AND polymorphism AND Met, and MICA AND polymorphism AND Val. Moreover, we exchanged polymorphism by SNP, Met by methionine, and Val by valine. We identified 17 publications, in which an association of the MICA-129Met/Val dimorphism with a disease or disease complication has been investigated. One study in Chinese language (24) appeared to be not independent of a larger study published in English (25). Thus, we analyzed 16 independent studies published between 2005 and 2015 (Table S1 in Supplementary Material). Three studies are small with less than 100 cases. All others are of a medium size with more than 100 but less than 1,000 patients included, and most studies used a case–control design.

Eight studies investigated associations with autoimmune diseases, i.e., ankylosing spondylitis (AS) (26), rheumatoid arthritis (RA) (27–29), inflammatory bowel disease (IBD) (25, 30) [including ulcerative colitis (UC) and Crohn’s disease], systemic lupus erythematosus (SLE) (28), type I diabetes (31), latent autoimmune diabetes in adults (LADA) (31), and psoriasis (32). In one study, the *MICA-129* SNP has not been determined directly. Instead, the SNP rs1051794 was typed and reported to be in complete linkage disequilibrium with the rs1051792 (27). Five studies reported on malignancies, i.e., nasopharyngeal cancer (33), hepatitis B virus (HBV)-induced hepatocellular carcinoma (HCC) (34), cutaneous malignant melanoma (35), and relapse of malignancy after hematopoietic stem cell transplantation (HSCT) (36, 37). Three studies investigated infections or their complications, i.e., HBV infection and HBV-induced HCC (34), left ventricular systolic dysfunction (LVSD) in chronic Chargas heart disease (38), and ocular toxoplasmosis (39). One study investigated an association of the MICA-129Met/Val dimorphism with recurrent miscarriage (40). The two studies on HSCT (36, 37) investigated besides relapse also other outcomes including graft-versus-host disease (GVHD).

Three studies, on recurrent miscarriage (40), ocular toxoplasmosis (39), and malignant melanoma (35), failed to demonstrate an association with the SNP. Thus, 81% of the studies showed an association at least for a subgroup, e.g., juvenile AS, whereas in all patients with AS, the association was dependent on HLA-B27 (26), or a sub-phenotype, e.g., severe LVSD (38). However, we must assume that other negative association studies have not been published. In seven studies, a *MICA-129* allele and the corresponding homozygous genotype were both associated with a disease risk (25, 28, 29, 31, 32, 34, 38). The odds ratio (OR) was then always higher for the genotype than the allele. In six studies, the *Met* allele and/or the *Met/Met* genotype were found to be associated with a risk, including autoimmune diseases [juvenile AS (26), UC (30), SLE (28), and psoriasis (32)], a malignancy (HBV-induced HCC) (34), and a complication of an infection (severe LVSD in chronic Chargas disease) (38). In three studies, the *Val* allele and/or the *Val/Val* genotype has been identified to confer a risk for autoimmune diseases [including RA (27), UC (25), and diabetes (31)] and for nasopharyngeal carcinoma (NPC) (33). Moreover, rheumatoid factor (RF) positivity in RA patients has been associated with the *Val* allele and the *Val/Val* genotype (29). In the studies on HSCT, different outcomes showed different associations. In one study (36), the *Met/Met* genotype was associated with an increased risk of relapse and the *Val/Val* genotype with an increased risk of chronic GVHD. In our recent study (37), the *Met/Met* genotype conferred a risk of acute GVHD, whereas having *Met* alleles reduced the risk to die from acute GVHD. Overall, the *Val* allele was associated with a higher mortality after HSCT (37).

**Figure 1:**
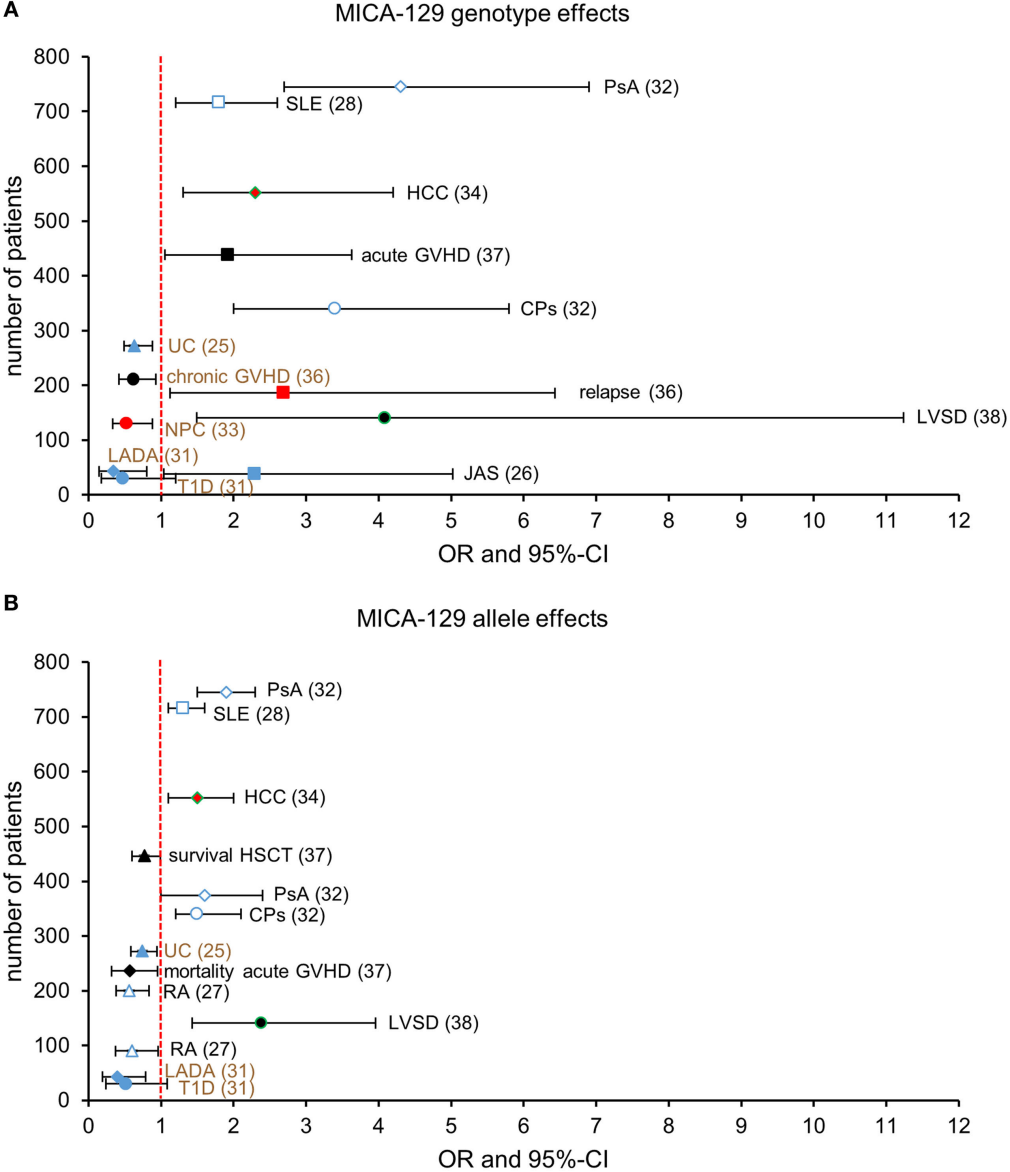
**Reported associations of the homozygous *MICA-129* genotypes (A) and the *MICA-129* alleles (B) with health risks [odds ratio (OR) > 1] or advantages (OR < 1)**. Shown are ORs with 95% confidence intervals (CI) or hazard ratios reported by Boukouaci et al. (36) and Isernhagen et al. (37) (overall survival) in event-time data. The number of patients analyzed in the studies is indicated at the *y*-axis. Studies reporting on autoimmune diseases are shown by open and closed blue symbols and malignancies by red symbols; studies reporting complications of infections (LVSD, Chargas disease; HCC, hepatitis B virus infection) are shown by green frames, and others are displayed by black symbols. The investigated diseases or complications and the references for the studies are indicated. **(A)** *MICA-129Met/Met* genotype effects are directly displayed. For studies that reported *MICA-129Val/Val* genotype effects [chronic GVHD (36), NPC (33), UC (25), RA (27), T1D, and LADA (31), indicated by brown font], the graph displays the corresponding effect of the pooled *MICA-129 Met/Met* and *MICA-129Met/Val* genotypes to allow for a direct comparison. **(B)** *MICA-129Met* allele effects are directly displayed. For studies that reported *MICA-129Val* allele effects [UC (25), T1D, and LADA (31), indicated by brown font], the graph displays the corresponding effect of the *MICA-129Met* allele; ORMet = 1/ORVal and 95%-CIMet = (1/CIVal, upper, 1/CIVal, lower). Abbreviations: CPs, cutaneous psoriasis; GVHD, graft-versus-host disease; HCC, hepatocellular carcinoma; JAS, juvenile ankylosing spondylitis; LADA, latent autoimmune diabetes in adults; LVSD, left ventricular systolic dysfunction; NPC, nasopharyngeal carcinoma; PsA, psoriatic arthritis; RA, rheumatoid arthritis; SLE, systemic lupus erythematosus; T1D, type 1 diabetes; UC, ulcerative colitis.

The results of these disease association studies do not allow for a simple unifying interpretation, such as the high-affinity MICA-129Met variant being associated with an activation of the immune system resulting in a lower risk of infections and cancer but higher risk of autoimmunity (Figures 1A,B). Autoimmune diseases are associated with both variants even within the same disease entity. UC, e.g., has been associated with the *Met/Met* genotype in a small study from Spain (30) but with the *Val* allele and *Val/Val* genotype in a larger study from China (25). RA has been associated with the *Val* allele in a study from France and Germany (27), but no association was found in cohorts from Japan (28) and Tunisia (29). Notably, a role of the NKG2D pathway has been reported for the pathogenesis of RA (41) and SLE (42), although this has not been linked to polymorphisms. Juvenile AS has been associated in a small study with the *Met/Met* genotype (26), and a larger sequencing study identified the MICA*007:01 allele that encodes a methionine at position 129 as a risk allele for AS in both Caucasian and Han Chinese populations (43). However, MICA*019, encoding a valine-129, has been identified as the major risk allele in Han Chinese (43). Malignancies were found to be associated with *Val/Val* genotype in the case of NPC (33) but with the *Met/Met* genotype in the case of relapse after HSCT (36). These different associations could suggest that the observed associations are random or dependent on the population studied. However, since the MICA-129Met/Val dimorphism is functional, it could also indicate that we need to better understand this function to predict its consequences in the pathophysiology of different diseases in various populations, which might be exposed to different interfering environmental factors. This assumption is supported by genome-wide association studies (GWAS), which have assigned disease risks for NPC (44), HCC (45, 46), cervical cancer (47, 48), and asthma (49) or advantages, such as HIV long-term non-progression (50) to the *MICA* gene region in an unbiased manner.

## Functional Consequences of the MICA-129Met/Val Dimorphism

**Figure 2:**
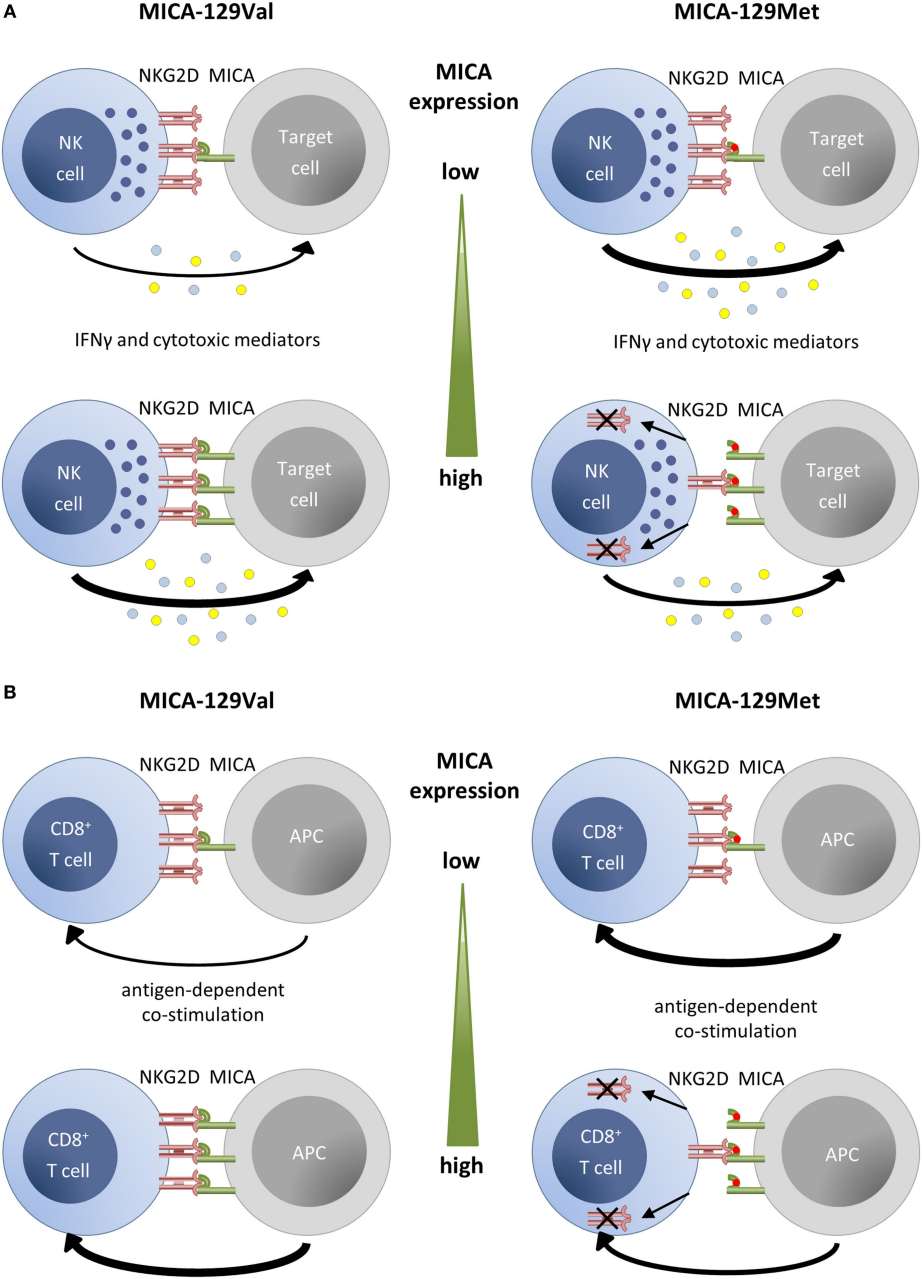
**Summary of functional effects of MICA-129 variants depending on expression intensity**. **(A)** For target cells expressing the MICA-129Val variant, the degree of natural killer (NK) cell cytotoxicity and IFN-γ production increases steadily with the MICA expression intensity. Augmented expression of the high-affinity MICA-129Met isoform, in contrast, has none or even a negative effect on these NK cell functions due to a rapid downregulation of NKG2D on NK cells. **(B)** Antigen-dependent costimulation of CD8^+^ T cells with the MICA-129Met variant allows for an earlier antigen-dependent activation than costimulation with the MICA-129Val variant. However, the downregulation of NKG2D in response to MICA-129Met ligands impairs any subsequent NKG2D-dependent costimulation and T cell activation. The downregulation of NKG2D on CD8^+^ T cells is augmented with MICA-129Met expression intensity. The figure is reproduced from Isernhagen et al. (37).

It has been shown by Steinle and colleagues that MICA-129Met isoforms bind NKG2D with high affinity in contrast to MICA-129Val isoforms that bind with low affinity (22). Yoshida and colleagues combined the MICA-129Met variant with the A9 variant of a microsatellite polymorphism in the transmembrane (TM) region and the MICA-129Val variant with the A5-TM variant in GST-fusion proteins (28). NK92MI cells showed a reduced NKG2D expression and killed K562 cells less efficiently when exposed to the MICA-129Met-A9-TM variant, but IFN-γ production was increased (28). We recently studied the consequences of binding of the two MICA-129 variants to NKG2D on primary NK cells and CD8^+^ T cells using cell lines transfected with expression constructs and recombinant Fc-fusion proteins differing only in amino acid 129 (37, 51). The recombinant MICA-129Met variant stimulated a stronger phosphorylation of SRC family kinases in NK cells than the MICA-129Val variant. Subsequently, the MICA-129Met ligand triggered more degranulation and IFN-γ production than the MICA-129Val ligand (Figure 2A). We then exposed NK cells to target cells expressing different amounts of the MICA-129 variants. The extent of degranulation and IFN-γ secretion correlated positively with the MICA expression intensity on the target cells but only for the MICA-129Val isoform. The expression intensity of the MICA-129Met isoform, in contrast, had either none or even a negative effect on the extent of degranulation, target cell killing, and IFN-γ release (37). On CD8^+^ T cells, the MICA-129Met isoform induced an earlier costimulatory activation than the MICA-129Val isoform (Figure 2B). Importantly, the MICA-129Met ligand induced also a stronger downregulation of NKG2D on both NK and CD8^+^ T cells than the MICA-129Val ligand. This downregulation of NKG2D impaired the capability of NK and CD8^+^ T cells to receive signals *via* NKG2D (37). Thus, MICA-129Met ligands, which elicit strong NKG2D responses, stimulate in parallel a robust negative feedback signal by downregulation of NKG2D that limits the initially stronger effects of MICA-129Met ligands. These data show that the biological effect of the MICA-129Met/Val dimorphism changes with the MICA expression intensity. Variant MICA-129Met triggers more NKG2D signals at low expression intensities, whereas variant MICA-129Val elicits more NKG2D effects at high expression, at which the MICA-129Met variant already downregulates NKG2D leading to impaired function. Thus, the biological effect of the SNP can hardly be predicted without information on the expression intensity of MICA.

It is known that expression intensities vary for certain *MICA* alleles (52, 53). The *G* allele of the SNP at -1878 (rs2596542) in the promoter region of the *MICA* gene region, e.g., was found to have a higher transcriptional activity (54). Biological effects of the MICA-129Met/Val dimorphism can be expected to be modified by polymorphisms affecting *MICA* gene expression. We have investigated whether the Met/Val dimorphism itself affects MICA expression. In transfected cells, more of the MICA-129Met variant was retained in intracellular compartments (51). A similar alteration of the intracellular transport has been described for *MICA-A5.1* variants (55). Thus, the combination of polymorphisms affecting transcription and intracellular transport of MICA could modify the effect of the Met/Val dimorphism.

Another important aspect of MICA is the generation of soluble MICA (sMICA) by proteolytic shedding. sMICA can induce NKG2D downregulation (56, 57) resulting in tumor immune escape (58). Some *MICA* polymorphisms have been reported to affect the amounts of sMICA in sera of patients including the SNP at -1878 (rs2596542) in the promoter region (34, 45, 59) that affects transcription (54), a microsatellite in exon 5 encoding the TM region (60, 61), and the MICA-129Met/Val dimorphism. In patients with UC, the *MICA-129Val/Val* genotype was associated with higher sMICA serum levels (25), and the *MICA-129Val* allele was also associated with higher sMICA serum levels in HBV patients and controls (34). In transfected cells, we found that the MICA-129Met isoform was more susceptible to shedding than the MICA-129Val isoform (51). However, due to the intracellular retention of the MICA-129Met variant (51), less sMICA might appear in sera (25, 34). Notably, intracellular retention and preferred shedding both appear to limit the expression of the high-affinity MICA-129Met isoform at the plasma membrane.

## MICA-129Met/Val Disease Associations in View of Biological Functions

Recent data on the MICA-129Met/Val variation demonstrate the complexity of the functional consequences of this exchange of a single amino acid (37, 51). There are several layers of this complexity, which are as follows: (1) the function of the variant is not constant but dynamic (37); it depends on the MICA expression intensity, and the direction of the biological effect can invert for the MICA-129Met variant at higher expression. (2) Epistatic effects must be expected for this SNP as polymorphisms affecting the expression of MICA will modify the functional effects of the MICA-129Met/Val isoforms. Moreover, the expression intensity of NKG2D can be modified by SNPs in the *KLRK1* gene (62) and those might interact with the *MICA-129* variants. Other genes within the NKG2D pathway including other ligands might also show epistatic effects (63). (3) MICA can target NKG2D on several cell types, and biological effects on different cell types might be synergistic or antagonistic. An activation of NK cells and a costimulation of CD8^+^ T cells both can promote antitumor immunity. By contrast, a strong activation of NK cells might polarize an immune response to a Th_1_ reaction and reduce the risk to develop a Th_2_-mediated autoimmune disease. (4) Additional factors, such as sMICA or anti-MICA antibodies (36) that might neutralize sMICA, have been shown to be functionally important and have been determined in some of the disease association studies (25, 34, 36).

Currently, we mostly have not sufficient clinical and biological information to interpret the MICA-129Met/Val disease association studies in view of the complex function of this polymorphism. However, the two HSCT studies do provide more information and illustrate the clinical effects of the MICA-129Met/Val dimorphism as explained previously in detail (37). In our study (37), the homozygous carriers of *Met* alleles had an increased risk to experience acute GVHD, possibly due to immediate strong effects of MICA-129Met variants on NKG2D signaling. Having at least one *Met* allele reduced the risk to die from acute GVHD likely due to a rapid downregulation of NKG2D on alloreactive CD8^+^ T cells mediated by engagement of a high-affinity MICA-129Met variant. Carrying a *MICA-129Met* allele increased in consequence the chance of survival in all patients and in patients receiving a *MICA-129*-matched graft (37). Boukouaci and colleagues reported an increased risk of chronic GVHD for recipients with the *Val/Val* genotype, whereas the *Met/Met* genotype was associated with the risk of relapse (36). Sustained NKG2D-mediated activation of alloreactive CD8^+^ T cells would be expected if only MICA-129Val variants are present that fail to efficiently downregulate NKG2D, and this could increase the risk of chronic GVHD but reduce the risk of relapse. Thus, the different risk associations reported in the two studies are not arguing against the relevance of the MICA-129 dimorphism for the outcome of HSCT. The principal relevance of the NKG2D pathway for HSCT is further emphasized by studies showing an effect of the genotype of the NKG2D ligand *RAET1L* (64) and NKG2D itself (65) on the survival of patients. Moreover, matching for *MICA* alleles (66–69) and specifically for the *MICA-129* polymorphism (70) is beneficial in HSCT. The huge effect of *MICA-129* matching appears hardly explainable solely by the avoidance of a potential minor histocompatibility antigen. A “tuning” of the threshold of NKG2D signaling toward the affinity of NKG2D ligands present in an individual (52) and disturbance of this balance by mismatching could be considered as an alternative explanation.

Despite the functional relevance of the *MICA-129* SNP, it cannot be excluded that some of the associations reported are random or caused by linkage disequilibrium with classical HLA genes. The association of MICA-129 with psoriasis (32) has been disproven in large GWAS cohorts (71). However, associations with NPC (33) and HCC (34) are supported by GWAS data pointing to the *MICA* gene region (44–46).

## Conclusion

Information on functional consequences of a polymorphism is indispensable for understanding disease associations. The variation in the disease associated allele or genotype of *MICA-129* in the published studies must not indicate random associations. For *MICA-129*, the biological function can change with expression intensity, epistatic interactions can be expected, the effect on different lymphocytes can vary, and modifying factors, such as sMICA, have to be considered. Notably, as expected for a functional SNP with a minor allele (*MICA-129Met*) frequency ranging from 48% in Africans to 30% in Asians (72), and being even the major allele reported in one of the analyzed studies (26), both alleles appear to confer advantages and disadvantages in specific situations suggesting balancing evolution of the *MICA* alleles. Since the MICA-129 dimorphism is considered as decisive for distinguishing low- and high-affinity variants (22), the frequency of alleles encoding high-affinity MICA variants is expected to match the frequency of the MICA-129Met variant. However, other *MICA* polymorphisms and their interaction need to be studied further (73).

In future studies, the MICA-129Met/Val dimorphism should be analyzed in larger cohorts. Detailed clinical information would help to understand why associations might differ in cohorts. Additional biological information should be obtained in parallel to genetic data. Most important would be data on MICA expression intensities in relevant tissues at relevant time points. Due to the complexity of MICA-129Met/Val effects, this polymorphism is unlikely to become a simple genetic biomarker for prediction of disease risks. However, it still may provide highly important information. We found that *Val/Val* genotype carriers undergoing HSCT specifically profited from a treatment with antithymocyte globulin to deplete T cells (37). This might be explained by a lack of a high-affinity MICA variant that efficiently downregulates NKG2D on alloreactive donor CD8^+^ T cells. Moreover, the *MICA-129* dimorphism might be relevant when considering therapies aiming at upregulation of MICA on tumor cells to sensitize them for NK cells (74, 75). Increasing the expression of MICA-129Met variants could result in opposite effects than intended.

## Author Contributions

RD searched the literature; RD and AI interpreted the functional data; RD and DM interpreted the genetic association data; RD drafted the manuscript; AI, DM, and HB commented the draft; and all the authors approved the final version.

## Conflict of Interest Statement

The authors declare that the research was conducted in the absence of any commercial or financial relationships that could be construed as a potential conflict of interest.
