# Supplementary material for: Impact of the MICA-129Met/Val Dimorphism on NKG2D-Mediated Biological Functions and Disease Risks
Source: Front Immunol. 2016 Dec 12;7:588. doi: 10.3389/fimmu.2016.00588 (PMC5149524; doi:10.3389/fimmu.2016.00588)
Supplement: Supplementary file 1 [file table_1.pdf]

Supplementary Material

Impact of the MICA-129Met/Val dimorphism on NKG2D-mediated biological functions and disease risks

Antje Isernhagen, Dörthe Malzahn, Heike Bickeböller, Ralf Dressel

Supplementary Table 1: MICA-129Met/Val disease association studies.

Study and reference	Population	Controls	Met allele frequency in controls	Disease	Patients	Risk allele	OR	95%-CI	p-value[a]	Risk genotype	OR	95%-CI	p-value[a]
1. Amroun 2005 (26)	Algeria	76	62 %	Ankylosing spondylitis	129	Met	2.57	1.29-5.1	Pc=0.006	Met/Met			P=0.0001 (HLA-B27 dependent)
				Juvenile ankylosing spondylitis (<15 years)	38					Met/Met	2.29	1.04-5.02	P=0.03 (HLA-B27 independent)
2. Boukouaci 2009 (36)	France		27 % (donors)	HSCT (with HLA-identical sibling donor)	211								
				occurrence of chronic GVHD	100 events, 111 censored					Val/Val	1.61	1.08-2.40	P=0.019
				relapse of malignancy	41 events, 145 censored					Met/Met	2.69	1.12-6.43	P=0.02
3. Douik 2009 (33)	Tunisia	180	27 %	Nasopharyngeal carcinoma	130	–			ns	Val/Val	1.87	1.14-3.04	Pc=0.02
4. Kirsten 2009 (27) (indirect LD with rs1051794)	France (family trios)	400	33 %	Rheumatoid arthritis	200	Val	0.56 (Met)	0.38-0.83	P=0.003				
	Germany (case-control)	182	33 %	Rheumatoid arthritis	90	Val	0.60 (Met)	0.37-0.96	P=0.032				
5. López-Hernández 2010 (30)	Spain	154	38 %	Inflammatory bowel disease	88	–			ns				
				Crohn's disease	59	–			ns				
				Ulcerative colitis	29	–			ns	Met/Met			Pc=0.028
6. Yoshida 2011 (28)	Japan	351	29 %	Systemic lupus erythematosus	716	Met	1.3	1.1-1.6	Pc=0.01	Met/Met	1.8	1.2-2.6	Pc=0.02
				Rheumatoid arthritis	327	–			ns	–			ns

Study and reference	Population	Controls	Met allele frequency in controls	Disease	Patients	Risk allele	OR	95%-CI	p-value[a]	Risk genotype	OR	95%-CI	p-value[a]
7. Zhao 2011 (25)	China	560	28 %	Ulcerative colitis	272	Val	1.35	1.06-1.72	P=0.015	Val/Val	1.59	1.14-2.05	P=0.005
				extensive v. distal UC	78 v. 194					Met carrier	0.59 (Val/ Val)	0.32-0.94	P=0.03
				severe v. other UC	34 v. 238					Val/Val	0.43 (Met carrier)	0.19-0.97	P=0.04
8. Raache 2012 (31)	Algeria	75	35 %	Type I diabetes	30	Val	1.92	0.92-4.21[b]	Pc=0.034	Val/Val	2.13	0.83-5.57[b]	Pc=0.043
				LADA	43	Val	2.51	1.27-5.18	Pc=0.002	Val/Val	2.94	1.25-7.02	Pc=0.003
9. Pollock 2013 (32)	Canada (Toronto)	547	27 %	Cutaneous psoriasis	340	Met	1.5	1.2-2.1	P<0.001	Met/Met	3.4	2.0-5.8	P<0.0001
				Psoriatic arthritis	745	Met	1.9	1.5-2.3	P<0.0001	Met/Met	4.3	2.7-6.9	P<0.0001
	Canada (St. John's)	115	26 %	Psoriatic arthritis	374	Met	1.6	1.0-2.4	P=0.035	–			ns
10. Tong 2013 (34)	Vietnam	418	37 %	HBV infection	552	–			ns	–			ns
				HCC v. non-HCC	166 v. 386	Met	1.5	1.1-2.0	P=0.009	Met/Met	2.3	1.3-4.2	P=0.006
11. Achour 2014 (29)	Tunisia	123	25 %	Rheumatoid Arthritis	142	–			ns	–			ns
				RF+ v. RF-	89 v. 38	Val	2.35	1.3-4.26	Pc=0.021	Val/Val	3.49	1.58-7.73	Pc=0.0095
12. Hizem 2014 (40)	Tunisia	334	27 %	Recurrent miscarriage	312	–			ns	–			ns
13. Ayo 2015 (38)	Brazil			LVSD in chronic Chargas heart disease	189								
				severe LSVD v. no LSVD	48 v. 93	Met	2.38	1.43-3.96	P=0.001	Met/Met	4.09	1.49-11.24	P=0.007
14. Ayo 2015 (39)	Brazil			Ocular Toxoplasmosis	297								
				presence v. absence	148 v. 149	–			ns	–			ns
15. Campillo 2015 (35)	Spain	200	36 %	Cutaneous malignant melanoma	233	–			ns	–			ns
16. Isernhagen 2015 (37)	Germany		32 % (donors)	HSCT	452								
				occurrence of acute GVHD	246 v. 192					Met/Met	1.92	1.05-3.63	P=0.0371
				mortality due to acute GVHD	44 v 202	Val	0.57 (Met, additive)	0.32-0.95	P=0.0400				
				occurrence of chronic GVHD	138 v. 302	–			ns				
				relapse of malignancy	86 v. 366	–			ns				
				overall survival	141 events, 305 censored	Val	0.77 (Met, additive)	0.60-0.99	P=0.0445				

Abbreviations: GVHD, graft versus host disease; HBV, hepatitis B virus; HCC, hepatocellular carcinoma; HLA, human leukocyte antigen; HSCT, hematopoietic stem cell transplantation; LADA, latent autoimmune diabetes in adults; LD, linkage disequilibrium; LSVD; left ventricular systolic dysfunction; RF, rheumatoid factor; UC, ulcerative colitis.

[a]P denotes nominal p-values. Pc denotes Bonferroni corrected p-values. Effect strengths with 95% confidence intervals (95%-CI) are odds ratio's (OR) except for Boukouaci et al. 2009 (36) and Isernhagen et al. 2015 (37) (overall survival) who estimated hazard ratio's in event-time data.

[b]As stated by Raache et al. 2012 (31).
